# MicroRNA Mediated Chemokine Responses in Human Airway Smooth Muscle Cells

**DOI:** 10.1371/journal.pone.0150842

**Published:** 2016-03-21

**Authors:** Mythili Dileepan, Anne E. Sarver, Savita P. Rao, Reynold A. Panettieri, Subbaya Subramanian, Mathur S. Kannan

**Affiliations:** 1 Department of Veterinary and Biomedical Sciences, University of Minnesota, St. Paul, Minnesota, United States of America; 2 Surgery, University of Minnesota, Minneapolis, Minnesota, United States of America; 3 Department of Medicine, University of Pennsylvania, Philadelphia, Pennsylvania, United States of America; Central Michigan University School of Medicine, UNITED STATES

## Abstract

Airway smooth muscle (ASM) cells play a critical role in the pathophysiology of asthma due to their hypercontractility and their ability to proliferate and secrete inflammatory mediators. microRNAs (miRNAs) are gene regulators that control many signaling pathways and thus serve as potential therapeutic alternatives for many diseases. We have previously shown that miR-708 and miR-140-3p regulate the MAPK and PI3K signaling pathways in human ASM (HASM) cells following TNF-α exposure. In this study, we investigated the regulatory effect of these miRNAs on other asthma-related genes. Microarray analysis using the Illumina platform was performed with total RNA extracted from miR-708 (or control miR)-transfected HASM cells. Inhibition of candidate inflammation-associated gene expression was further validated by qPCR and ELISA. The most significant biologic functions for the differentially expressed gene set included decreased inflammatory response, cytokine expression and signaling. qPCR revealed inhibition of expression of *CCL11*, *CXCL10*, *CCL2* and *CXCL8*, while the release of CCL11 was inhibited in miR-708-transfected cells. Transfection of cells with miR-140-3p resulted in inhibition of expression of *CCL11*, *CXCL12*, *CXCL10*, *CCL5* and *CXCL8* and of TNF-α-induced CXCL12 release. In addition, expression of *RARRES2*, *CD44* and *ADAM33*, genes known to contribute to the pathophysiology of asthma, were found to be inhibited in miR-708-transfected cells. These results demonstrate that miR-708 and miR-140-3p exert distinct effects on inflammation-associated gene expression and biological function of ASM cells. Targeting these miRNA networks may provide a novel therapeutic mechanism to down-regulate airway inflammation and ASM proliferation in asthma.

## Introduction

Several recent reports have provided evidence that airway smooth muscle (ASM) has strong pro-inflammatory and immunomodulatory functions [1–4]. These properties of ASM are mediated through its synthetic function as well as through expression of a variety of cell-surface molecules, integrins [5–7], and Toll-like receptors [8, 9]. During acute airway inflammation, mediators and cytokines released from structural and inflammatory cells alter ASM contractile function [10–13]. However, during persistent airway inflammation, cytokines and chemokines produced by inflammatory cells and ASM can cause ASM proliferation, leading to structural changes in the airways, often referred to as airway remodeling [14, 15]. During chronic airway inflammation, the immunomodulatory role of ASM may be more significant in establishing structural changes within the airways than its contractile function. In this context, recent reports show that ASM is capable of releasing cytokines such as IL-5 [16, 17], IL-6 [18, 19], IL-33 [20], TSLP [21], GM-CSF [22] and VEGF [23]; chemokines such as RANTES[16], Fractalkine [24], CCL11 [25], CXCL10[26–28], CXCL8[29]; adhesion molecules such as ICAM-1[30, 31], VCAM-1 [30, 32], CD44 [33] and LFA-1[34]; and growth factors such as IGF-1 [35, 36] and stem cell factor [37]. Cytokines released by immune cells recruited into the lungs during allergic inflammation may also stimulate ASM cells to alter the expression of proinflammatory genes in an autocrine or paracrine manner. There is also evidence for hypersecretion of chemokines both constitutively and in response to cytokines in ASM cells obtained from asthmatics than in cells from non-asthmatics [14, 38]. There is also increased chemotaxis of mast cells toward ASM cells from asthmatics both *in vivo* and *in vitro* [28, 39–41]. Other studies have examined the transcriptional regulation of expression of chemokine genes in human ASM cells (HASM) [29, 42]. While such transcriptional regulation of expression of chemokines is better understood, the post-transcriptional regulation is an emerging area of investigation. In this context, recent studies provide evidence for specific microRNAs in the regulation of ASM proliferation [43, 44], ASM phenotype [45] and airway inflammation [46, 47].

microRNAs (miRNAs) are small non-coding ~22nt RNAs that regulate gene expression by binding to the 3’-Untranslated Region (3’UTR) of target mRNAs to cause mRNA degradation and/or translational repression [48]. Since binding of miRNAs to target sequences is dependent on its ‘seed’ sequence, a single miRNA can potentially regulate a large number of genes. Specific miRNAs have already been discovered that regulate cellular functions such as differentiation, proliferation, and apoptosis. [48–50] Dysregulation of miRNA expression has been implicated in airway inflammation [48–50], but the specific miRNAs (miR-140-3p and miR-708) controlling inflammation have not previously been reported. In a recent report we identified miR-708 in the post-transcriptional regulation of expression of a cell-surface protein CD38 through two major signaling pathways [51]. Transfection of HASM cells with miR-708 causes the induction of phosphatase and tensin homolog (*PTEN*), which regulates PI3K/AKT signaling by decreasing Akt phosphorylation and interacts with members of the NF-κB signaling network. miR-708 also induces *DUSP-1*, a dual specificity phosphatase, leading to JNK MAPK dephosphorylation [51].

Our recent investigations also provided evidence for down-regulation of p38 MAP kinase and NF-κB activation in HASM cells following transfection with miR-140-3p [52]. The net effect of *PTEN* and *DUSP-1* induction as well as inhibition of MAP kinase and NF-κB activation in ASM cells by miRNAs should lead to modulation of key signaling pathways involved in inflammation and cell proliferation. There is evidence that the expression of several chemokine genes, the release of chemokines and cell proliferation in HASM cells are also regulated by these same signaling pathways [53–59].

In this study, we evaluated differentially expressed genes using microarrays and qPCR in HASM cells following miR-708 transfection and stimulation with the inflammatory cytokine TNF-α, with particular emphasis on the expression of cytokine/chemokine genes, other pro-inflammatory genes, and those reported to be involved in the asthmatic phenotype. Since many of these chemokines are involved in the recruitment of inflammatory cells such as eosinophils, basophils, mast cells and T lymphocytes into the airways during allergic airway disease, we measured their release from cells stimulated with the inflammatory cytokine TNF-α and following transfection with miR-708 or miR-140-3p.

## Materials and Methods

Ethics statement: Airway smooth muscle cells from human lungs were prepared in Dr. Panettieri’s laboratory at the University of Pennsylvania. Lung tissues were obtained from the National Disease Resource Interchange (NDRI) and its use was approved by the Institutional Review Board at the University of Pennsylvania and University of Minnesota. All donor tissue is harvested anonymously and de-identified and therefore the use of the cells does not constitute human subjects research. Primary ASM cells were isolated from deceased donors.

### Reagents

Reagents used in the current study: DMEM from GIBCO-BRL (Grand Island, NY); rh-TNF-α from R&D Systems (Minneapolis, MN); TRIzol, SuperScript III reverse transcriptase, Opti-MEM^®^ reduced serum medium and Lipofectamine^®^ RNAiMax transfection reagent from Invitrogen Life Technologies (Carlsbad, CA); Brilliant lll Ultra-Fast SYBR Green qPCR Master Mix from Agilent Technologies Inc (Santa Clara CA); control oligo (scrambled sequence mimic) and miR-708 mimic (mature miR-708 sequence: 5’-AAGGAGCUUACAAUCUAGCUGGG-3’; mature miR-140-3p sequence: 5′-UACCACAGGGUAGAACCACGG-3′) from Dharmacon (Lafayette, CO); Tris-base, glucose, HEPES and other chemicals from Sigma Chemical Co. (St. Louis, MO).

### Microarray sample preparation

ASM cells, derived from three de-identified healthy donors, used between 2-5^th^ passages, were seeded at 1.5 X 10^5^ cells/well and transfected with mimic or scrambled sequence mimic of miR-708 at 50 nM concentration [51]. We used the same concentration which was previously determined to be optimal to inhibit the expression of CD38 [51]. Cells that were growth arrested (24 h) after transfection, were induced with pro-inflammatory cytokines TNF-α at 10 ng/ml (24 h). Total RNA was harvested using PureLink RNA isolation kit according to the manufacturer’s instructions. Purity of the RNA was determined with a Nanometer 2000C for the ratios 260/280 and 260/230. For each condition (mimic or scrambled oligo treatment), 1000 ng of total RNA was subjected to microarray analysis. Genome-wide changes in gene expression in transfected cells were generated using Illumina human (HT-12) arrays and analyzed using BeadStudio version 3.1.1.

### Data Analysis

For statistical analysis and clustering, we used the Partek Genomics Suite software package (Partek Inc., St. Louis, MO, USA). We performed a paired *t*-test with donor ID and mimic/control as the nominal variables. Before comparison analysis and clustering, we filtered extremely low and non-variant genes out of the datasets. Significance cutoff filters were set at *P* < 0.05 and an expression change of at least 2-fold. For functional and pathway analyses we used Ingenuity Pathway Analysis (IPA) software (Qiagen, Redwood City, CA, USA). IPA employs a right-tailed Fisher exact test to calculate a *P* value corresponding to the probability that a biologic function not relevant to the input dataset is falsely identified as relevant. A Benjamini–Hochberg false discovery rate of 0.05 was used to correct such *P* values.

### Validation of genes by qPCR

As described in the “Microarray sample preparation”, total RNA was isolated and cDNA was prepared using reverse transcription kit from Invitrogen Life Technologies (Carlsbad, CA). cDNAs were subjected to qPCR analysis using Brilliant SYBR Green Master Mix and Stratagene Mx3000p qPCR system (Foster City, California, 94404). Primer sequences and conditions for the genes tested are provided in Table 1. The β-actin gene was used as a housekeeping gene to normalize the expressions of other genes.

**Table 1:** Primer Sequences.

Gene	primer sequences
**CXCL10**	F: 5'—3' GAACTGTACGCTGTACCTGCA
	R: 5'—3' TTGATGGCCTTCGATTCTGGA
**CXCL8**	F: 5'—3' ACTGAGAGTGATTGAGAGTGGAC
	R: 5'—3' AACCCTCTGCACCCAGTTTTC
**CCL5**	F: 5'—3' CAGTCGTCTTTGTCACCCGAA
	R: 5'—3' TCCCAAGCTAGGACAAGAGCA
**CCL2**	F: 5'—3' AGGTGACTGGGGCATTGAT
	R: 5'—3' GCCTCCAGCATGAAAGTCTC
**CXCL12**	F: 5'—3' TGCCAGAGCCAACGTCAAG
	R: 5'—3' CAGCCGGGCTACAATCTGAA
**CCL11**	F: 5'—3' CCCCAGAAAGCTGTGATCTTCA
	R: 5'—3' GGAGTTGGAGATTTTTGGTCCAGAT
**RARRES2**	F: 5'—3' GAGGGACTGGAAGAAACCCG
	R: 5'—3' CATGGCTGGGGATAGAACGG
**ADAM33**	F: 5'—3' GACCTAGAATGGTGTGCCAGA
	R: 5'—3' AGCCTGGC TTGTCACAGAAG
**CD44**	F: 5'—3' AGCATCGGATTTGAGACCTG
	R: 5'—3' GTCCACATTCTGCAGGTTCC
**β-Actin**	F: 5'—3' ACACTGTGCCCATCTACGAGG
	R: 5'—3' AGGGGCCGGACTCGTCATACT

### Chemokine Release assay

HASM cells were transfected with mimic or scrambled sequence mimic of miR-708 or miR-140-3p or were untransfected (control) as described in earlier publications [51, 52]. Cells were then growth arrested and treated with 10ng/ml TNF-α. Cell culture supernatants were collected at different time points ranging from 6–48 h. Collected supernatants were aliquoted and immediately stored at -80°C until assayed. Chemokines in the culture supernatants were quantified using ELISA kits from R&D system (Minneapolis, MN) according to the manufacturer’s instructions.

## Results

### Microarray results

**Fig 1:**
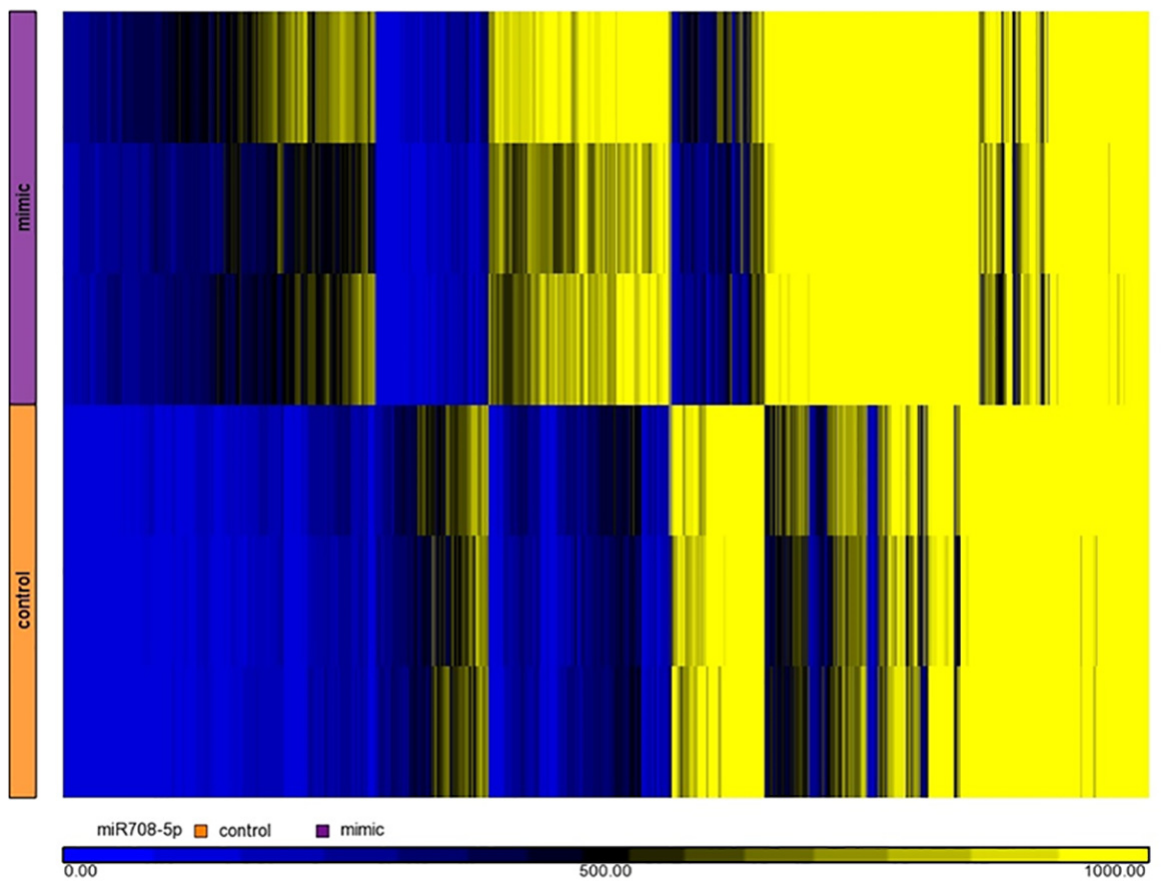
Heat map of mRNA microarray expression data. Purple bar indicates samples treated with the miR-708 mimic; orange bar indicates samples treated with a scrambled control. Sample rows are arranged in the same donor-order (i.e. donor 1 samples are rows 1 and 4).

**Fig 2:**
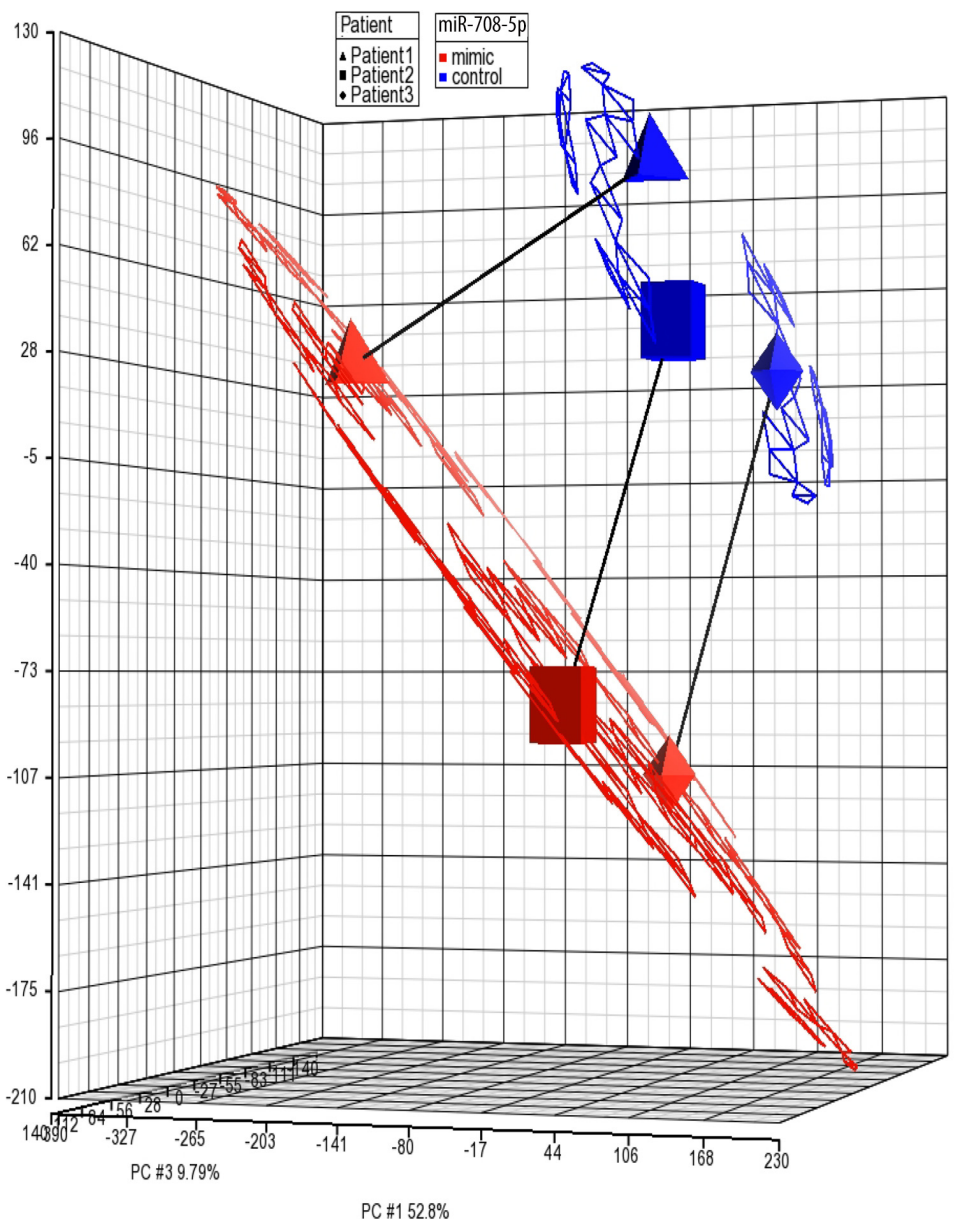
Principal Component Analysis. There is a clear primary separation of samples based on miR-708 mimic versus scrambled control. Secondary separation was by donor ID.

We performed a detailed analysis of the pattern of gene expression in HASM cells stimulated with TNF-α following transfection with miR-708. Gene expression results are shown as a heatmap (Fig 1). Visual inspection easily identifies differential patterns of expression between samples treated with a miR-708-5p mimic (purple bar) versus a scrambled control (orange bar). Principal Component Analysis (PCA) confirmed the mimic as the primary differential component (Fig 2). Our analysis found that 821 genes were differentially expressed (348 upregulated and 473 downregulated) in HASM cells transfected with a miR-708 mimic versus a scrambled control sequence (paired t-test, *P* < 0.05). Table 2 summarizes the differentially expressed chemokines/cytokines, transcription factors, extracellular matrix components, calcium signaling molecules, growth factors and other genes related to airway hyperresponsiveness. The complete list of genes is available as S1 Data.

**Table 2:** Differentially expressed genes in TNF-α-stimulated HASM cells following miR-708 mimic transfection.

	Gene ID	Fold change	Function
Inflammatory mediators			
Chemokines	*CXCL10*	-2.695	Chemoattracts mast cell
	*CCL8*	-5.38	Chemoattracts monocytes
	*CCL11*	-8.968	Chemoattracts Eosinophils
	*CXCL12*	-5.254	Chemoattractant T-lymphocytes & monocytes
	*CCL5*	-3.52	Chemoattracts Eosinophil
	*CCL2*	-3.21	Chemoattracts monocytes, fibrocytes and basophils
	*CXCL8*	-2.03	Chemoattracts neutrophils, basophils and T-cells
	*CXCL16*	-2.688	Scavenger receptor on macrophages
	*CXCL5*	-2.389	Activates neutrophils
	*CXCL9*	-2.375	Chemoattracts activated T-cells
	*CXCL11*	-2.151	Chemoattracts interleukin-activated T-cells
	*CXCL6*	-2.039	Chemoattracts neutrophil, granulocytes
Cytokines			
	*IL18BP*	-4.028	Inhibits the early TH1 cytokine response
	*IL6*	-1.832	Stimulates the differentiation of B-cells and acts as a myokine.
	*TNFSF13B*	-4.216	Stimulates B- and T-cell function
Genes associated with Extracellular Matrix			
	*VCAM1*	-20.59	Enhances leukocyte-endothelial cell adhesion and T cell inflammatory functions
	*COL3A1*	-9.808	Activates RhoA pathway
	*COL6A1*	-3.185	A major structural component of microfibrils
	*CD44*	-4.25	Increases airway hyperresponsiveness [80]; leads to inflammation [61, 81] by interacting with T-cell [33] and mast cell [67]; increases ASM cell proliferation [82]
	*THBS1*	-4.797	Increases IL-8 production [83]
	*MXRA5*	-4.581	Associates with matrix-remodeling protein
	*ADAMTS-1*	6.158	Increases FEV1 [84]
	*TAPBP*	-4.38	Increases antigen processing andassembly of MHC class I [85]
Transcription factors			
	*NFKB1*	-1.876	Regulates immune response
	*RELA*	-1.728	Regulates immune response
Calcium signaling			
	*CD38*	-2.286	Increases cell adhesion, signal transduction, AHR and calcium signaling.
	*BDKRB1*	-1.994	Increases chronic and acute inflammatory responses
	*FKBP10*	-2.198	Regulates [Ca2+]i dynamics
Growth factors and related genes			
	*IGFBP5*	-4.009	Prolongs the half-life of the IGFs
	*PDGFRL*	-4.258	Increases proliferation
	*EGFL6*	-3.886	Regulates cell cycle & induces proliferation
Airway hyper-responsiveness			
	*ACTG2*	-17.181	Increases muscle contraction
	*TAGLN*	-4.691	Increases calcium interactions and contractility
	*MYLK*	-2.12	Increases Smooth muscle contraction
	*PDE5A*	-2.50	Inactivates cGMP [86]
Genes associated with proliferation	*F2F7*	6.8	Anti-proliferative [87]
	*IL24*	11.6	Anti-proliferative [88]
	*COL1A1*	-7.474	Increases ASM cell proliferation [89]
	*DUSP6*	10.915	Decreases ASM cell proliferation
	*UBE2C*	18.96	Increases cell proliferation
	*CDC20*	16.459	Increases cell proliferation
	*ID1*	16.089	Increases cell proliferation
	*ANGPTL4*	12.633	Increases ASM cell proliferation [90]
	*CDK1*	5.387	Increases cell proliferation

### Functional and Pathway Analysis

The most significant biologic functions for this differential gene set included decreased inflammatory response, cytokine expression and signaling. In particular, many components of the IL-17 pro-inflammatory pathway were down-regulated. Multiple pathways and biologic functions related to cell cycle progression were predicted to be upregulated.

### miR-708 inhibits chemokine mRNA expression and other asthma related genes

**Fig 3:**
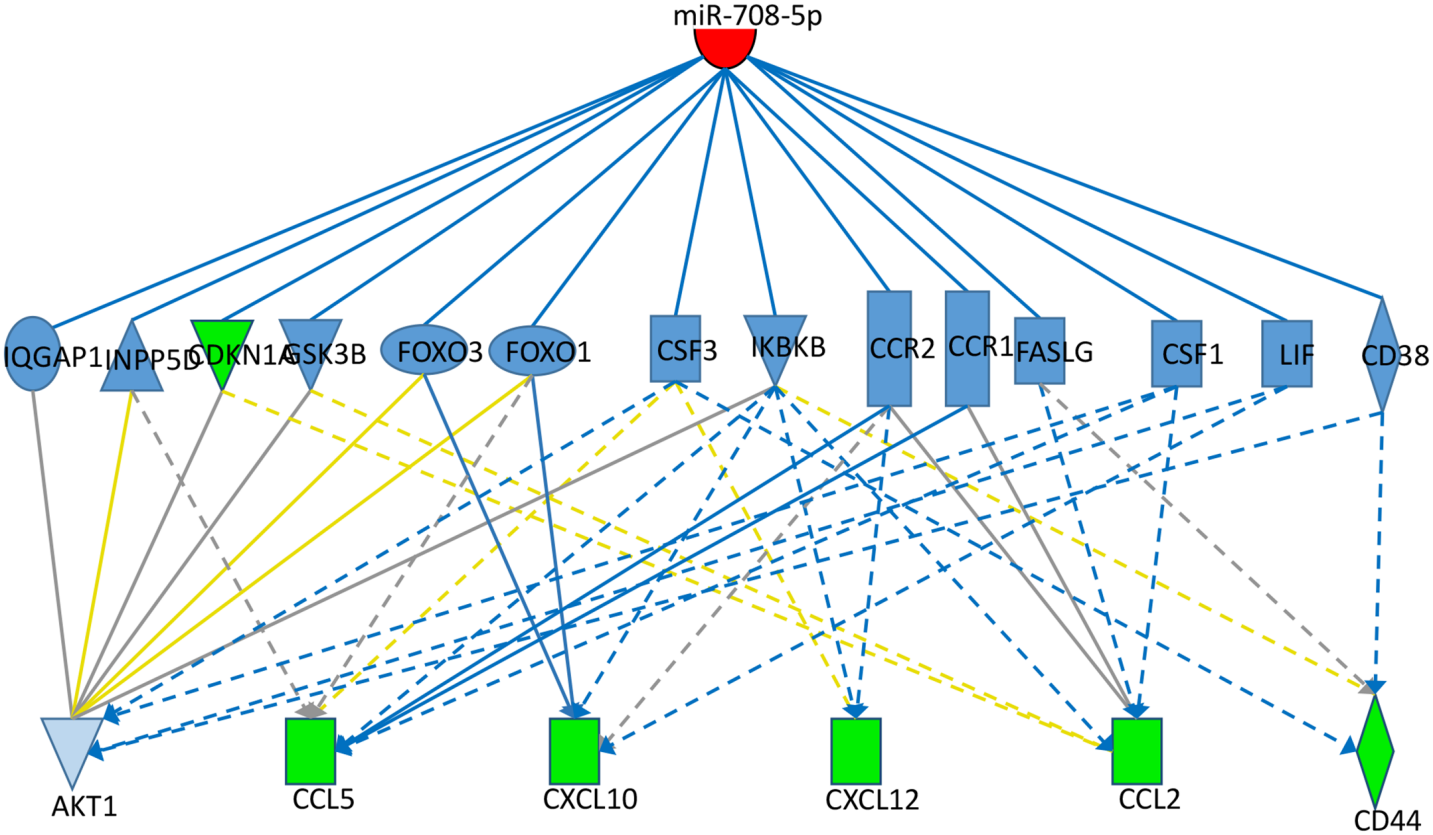
Network diagram. Potential regulatory pathways connecting miR-708 and down-regulated molecules of interest in HASM cells. Several chemokine genes were observed to be significantly down-regulated, particularly CD44 and CD38 (-3.23 and -2.287, respectively). Nodes are colored either by observed expression changes in the paired t-test (Green) or by predicted activation status (Blue = predicted inhibition) based on the assumption of increased miR-708-5p (Red). Potential relationships are indicated by solid (direct interaction) or dotted (indirect interaction) lines. Interaction lines are colored based on whether the predicted relationship leads to inhibition (Blue), leads to predicted activation (Yellow; but inconsistent with observed results), or effect was not able to be predicted (Gray).

**Fig 4:**
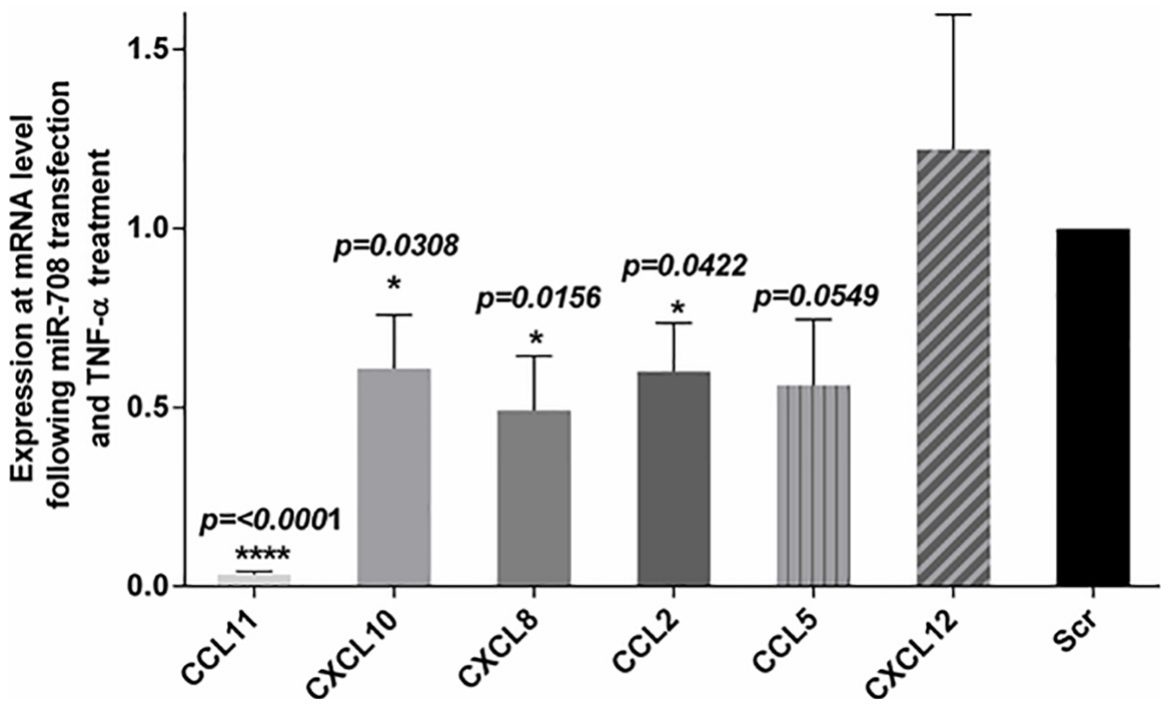
Downregulation of chemokine mRNA expression following miR-708 transfection. HASM cells derived from 3–5 donors were transfected with mimic or scrambled (Scr) sequence mimic of miR-708 followed by exposure to TNF-α (10ng/ml) to measure chemokine mRNA expression. Note the significant inhibition in the expression of CCL11, CXCL10, CXCL8 and CCL2 following miR-708 mimic transfection compared to expression in cells transfected with scrambled sequence. Data represents mean±SEM.

**Fig 5:**
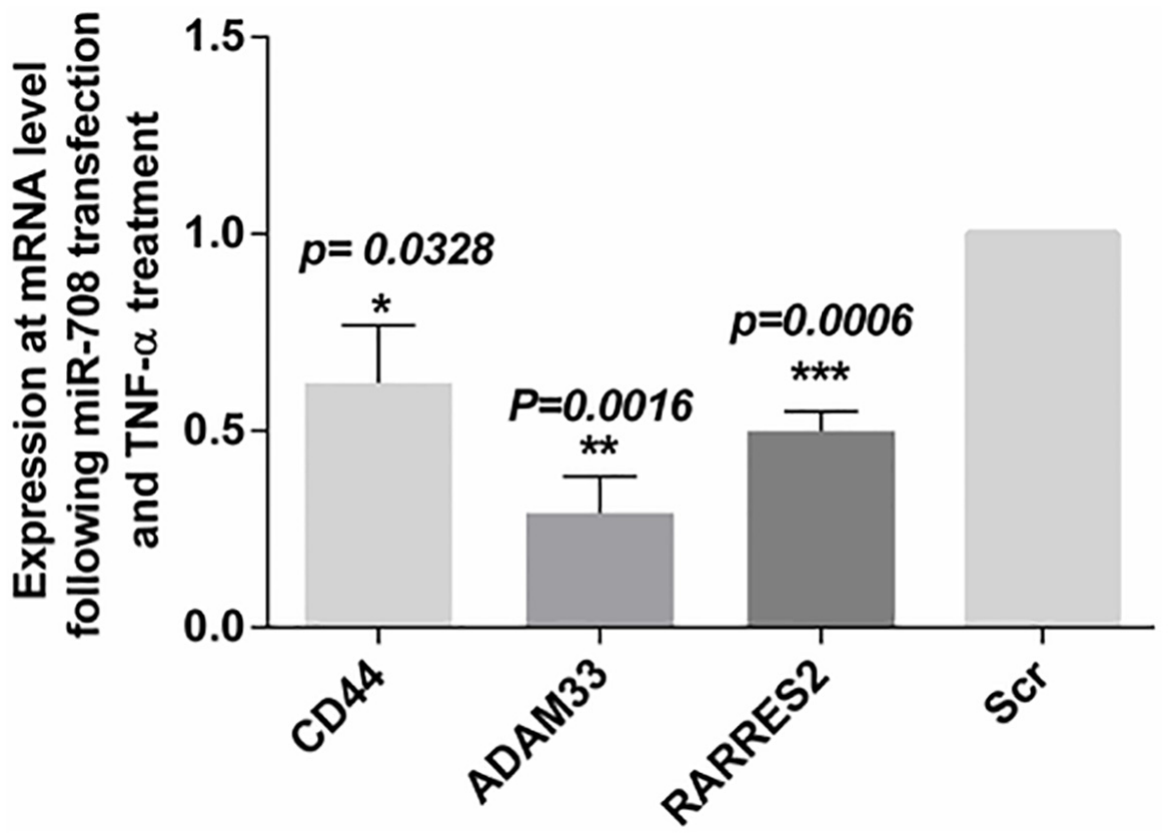
Down regulation of other ‘asthma related’ genes by miR-708. HASM cells obtained from 3–5 donors were transfected with mimic or scrambled sequence mimic (Scr) of miR-708 followed by treatment with TNF-α (10ng/ml/). Note significant inhibition of expression of CD44, ADAM33 and RARRES2 transcripts in miR-708 mimic-transfected cells compared to expression in scrambled sequence transfected cells. Data represents mean±SEM.

Results of gene expression analysis revealed significant down-regulation of expression of several chemokine genes as well as some genes associated with the asthmatic phenotype (Fig 3). Therefore, we used qPCR analysis to validate the microarray results of expression of these genes. HASM cells were transfected with miR-708-5p mimic oligonucleotides or the scrambled control and then treated with TNF-α. 24 hours following the addition of TNF-α, total RNA was collected from the cells and subjected to qPCR analysis. There was significant inhibition in the expression of chemokine genes *CCL11* (*P* <*0*.*0001*), *CXCL10* (*P = 0*.*0308*), *CCL2* (*P = 0*.*0422*) and *CXCL8* (*p = 0*.*0156*) (Fig 4) as well as other ‘asthma related’ genes such as *CD44* (*P = 0*.*0328)*, *ADAM33* (*P = 0*.*0016*) and *RARRESS2* (*P = 0*.*0006)* (Fig 5). On the other hand, the mRNA expression levels for chemokine genes *CCL5* (*P = 0*.*0549*) and *CXCL12* following transfection with the miR-708 mimic were not significantly different from expression in scrambled oligonucleotide-transfected cells (Fig 4).

### miR-708 transfection and release of chemokines

**Fig 6:**
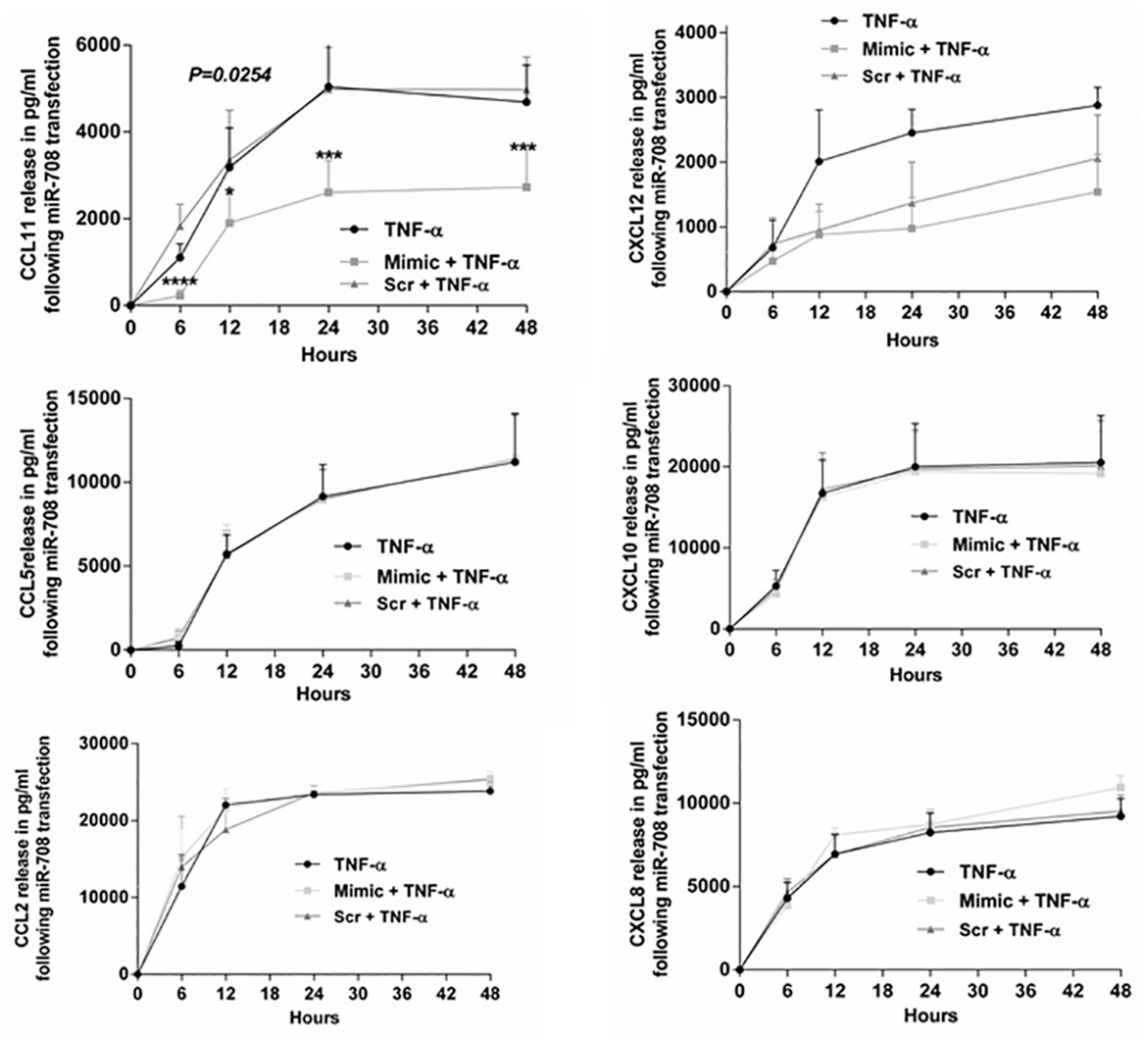
Chemokine release from HASM cells following miR-708 transfection. HASM cells from 3–6 donors were transfected with mimic or scrambled sequence mimic of miR-708 and treated with TNF-α (10ng/ml) following growth arrest of cells. Untransfected cells treated with TNF-α served as an additional control. Twenty hours later cell culture supernatants were collected for the measurement of chemokines. Note the release of CCL11 was significantly inhibited at every time point following miR-708 transfection when compared to scrambled sequence mimic transfection. Data represents mean±SEM.

To determine whether changes in the mRNA expression of chemokines were reflected in their protein expression, we measured their release in HASM cell culture supernatant following miR-708 mimic or scrambled sequence mimic transfection and TNF-α induction. As a control, we collected the culture supernatant from untransfected but TNF-α treated HASM cells. Of the chemokines that were assayed, only CCL11 release exhibited significant downregulation of release in mimic miR-708-transfected cells compared to release from cells transfected with the scrambled oligonucleotide or from control cells at all time points examined (Fig 6).

### miR-140-3p transfection and chemokine mRNA expression and release

**Fig 7:**
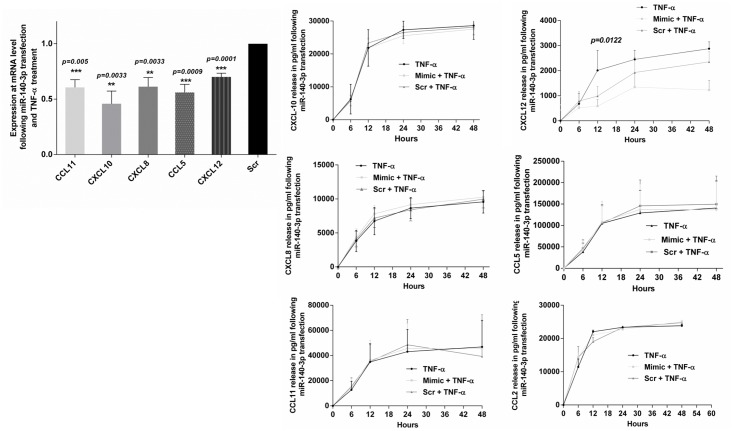
Chemokine mRNA expression and release from HASM cells following miR-140-3p transfection. HASM cells derived from 3–5 donors were transfected with mimic or scrambled (Scr) sequence mimic of miR-140-3p followed by exposure to TNF-α (10ng/ml) to measure chemokine mRNA expression and chemokine release. Note the significant inhibition in the expression of CCL11, CXCL10, CXCL8, CXCL12 and CCL5 following mimic transfection compared to expression in cells transfected with scrambled sequence. Note the release of CXCL12 was significantly inhibited following mimic transfection. Data represents mean±SEM.

The expression of many of the chemokine genes in HASM cells is regulated by signaling pathways that are downregulated by miR-140-3p. Therefore, we measured the expression and release of chemokines in response to TNF-α following miR-140-3p transfection. HASM cells were transfected with miR-140-3p mimic oligonucleotides or the scrambled control and then treated with TNF-α. Twenty four hours following the addition of TNF-α, total RNA was collected from the cells and subjected to qPCR analysis. There was significant inhibition in the expression of chemokine genes CCL11 (*p =* <*0*.*005*), CXCL12 (*p<0001*), CCL5 (*p<0*.*0009*), CXCL10 (*p = 0*.*0033*), CCL2 (*p = 0*.*0422*) and CXCL8 (*p = 0*.*0033*) (Fig 7). We measured the release of chemokines in HASM cell culture supernatant following miR-140-30 mimic or scrambled sequence mimic transfection and TNF-α induction. As a control, we collected the culture supernatant from untransfected but TNF-α-treated HASM cells. Of the chemokines that were assayed, only CXCL12 release exhibited significant down-regulation of release in mimic miR-140-3p-transfected cells compared to release from cells transfected with the scrambled miR-140-3p oligonucleotides or from control cells at all time points examined (Fig 7).

## Discussion

Using a transcriptomics-based approach, we investigated differentially expressed genes in HASM cells treated with TNF-α following miR-708 transfection compared to expression in cells transfected with the scrambled mimic oligonucleotides. This analysis revealed changes in the expression of several genes, including those for chemokines/cytokines, extracellular matrix proteins, transcription factors, calcium signaling molecules, growth factors, and genes associated with airway hyperresponsiveness. Several genes involved in cell cycle regulation were up-regulated, although the genes that block cell proliferation such as *E2F7*, *DUSP6* and *IL-24*, were also significantly upregulated. There was downregulation of expression of *JNK MAP* kinase which is involved in serum-induced ASM cell proliferation [60]. The changes in the expression of chemokine genes revealed in this approach were confirmed by qPCR. In addition, miR-708 also caused downregulation of expression of several ‘asthma-related’ genes such as *CD44* [33, 61], *ADAM33* [62, 63] and *RARRES2* [64–66]. Prior reports have shown that CD44 is involved in mast cell-ASM cell adherence through Type I collagen and this adherence is greater during airway inflammation as well as in ASM cells derived from asthmatics [67]. miR-140-3p transfection of HASM cells also resulted in inhibition of expression of chemokines that were sensitive to inhibition by miR-708, with the exception of *CXCL12*. However, chemokine release measurements revealed inhibition of release of CXCL12, but not the other chemokines.

In the present study, we examined the post-transcriptional regulation of expression of several inflammatory genes in HASM cells by miR-708. HASM cells express miR-708 and miR-140-3p constitutively and TNF-α causes a significant reduction in their expression [51, 52]. Furthermore, the constitutive expression of DUSP-1 and PTEN are also significantly downregulated following exposure to TNF-α [51]. Transfection with miR-708 in cells stimulated with TNF-α resulted in a significant augmentation of *PTEN* and *DUSP*-1 expression, with concomitant decreased activation of Akt and JNK MAP kinase, respectively [51]. The PI3 kinase/Akt and MAP kinase signaling mechanisms are involved in airway inflammation by activating transcription factors such as NF-κB and AP-1 [68–71]. Recent reports have shown that this signaling is involved in the hyperproliferative phenotype of ASM cells from asthmatics [54]. Our earlier study showed that miR-140-3p decreases the activation of p38 MAP kinase and NF-κB in HASM cells. The promoter regions of several chemokine genes contain binding sites for NF-κB and AP-1 as well as for other transcription factors [72–74]. Furthermore, TNF-α has been shown to induce the expression and release of cytokines/chemokines from HASM cells [25, 41, 42, 75, 76] including the chemokines that we have examined in this study. Although the mechanisms by which miR-708 decreased the expression of the chemokine genes that we examined are not addressed in this study, the 3’UTRs of *CXCL12* and CCL5 have predicted target sites for miR-708 indicating that this miRNA may directly target these transcripts. As well, miR-140-3p also has predicted binding sites at 3’UTR of CXCL12 and CXCL8. It is very likely that the inhibition of expression of chemokine genes following miR-708 or miR-140-3p transfection resulted from indirect mechanisms of decreased activation of transcription factors and MAP kinases as well as binding of miRNAs to cause translational repression and/or mRNA breakdown.

The miRNAs examined in this study had profound inhibitory effects on the chemokines involved in the recruitment of eosinophils, mast cells, T lymphocytes and fibrocytes. Chemokine release studies additionally revealed inhibition of release of CCL11 and CXCL12. It is interesting to note that our microarray results showed a high level of downregulation of *CXCL12* expression by miR-708, while the qPCR results did not show any change in *CXCL12* transcript levels following miRNA transfection. It is very likely that miR-708 may regulate transcription and release of some chemokines while it may have a dominant effect on release for others. It is also known that production of specific chemokines in ASM cells may involve unique signaling pathways and stimuli, as has been shown for CXCL10 release [41]. In this study, it was reported that in HASM cells exposed to TNF-α or IL-1β, CXCL10 production required JNK MAP kinase activation, while its release was induced by p38 MAP kinase activation. The results of the microarray analysis of differentially expressed genes and qPCR results confirmed selective downregulation of JNK MAP kinase expression by miR-708, with decreased JNK MAP kinase phosphorylation. This decreased JNK MAP kinase activation may be a mechanism involved in the inhibition of CCL11 release and expression in miR-708 transfected cells following exposure to TNF-α. It should be noted that among the chemokine transcripts examined, miR-708 transfection resulted in a profound inhibition of CCL11 expression while the inhibition of expression of other chemokine transcripts was modest and not of sufficient magnitude to be reflected in inhibition of release. Transfection of cells with miR-140-3p caused significant attenuation of expression of all the five chemokine genes examined, while exerting a selective inhibitory effect on the release of CXCL12 but not the other chemokines. The decreased p38 MAP kinase activation following miR-140-3p transfection noted in our previous studies [59] may be involved in the attenuation of CXCL12 release in response to TNF-α. Furthermore, these miRNAs may selectively inhibit the release of some chemokines but not others. Recent investigations have shown that stimulation of ASM cells with a mixture of cytokines causes significantly higher amounts of chemokine release than following exposure to individual cytokines [56, 59]. It should be emphasized that in our study chemokine release was measured from cells following miRNA transfection and growth-arrest, before stimulation with TNF-α. It will be interesting to examine release of chemokines in response to a mixture of cytokines following miRNA transfection.

In conclusion, this study demonstrates a profound anti-inflammatory effect of miR-708 and miR-140-3p in HASM cells stimulated with the inflammatory cytokine TNF-α. Specifically, targeting these miRNAs resulted in the down-regulation of expression of multiple different chemokines and the release of specific chemokines involved in the recruitment of inflammatory cells into the airways during allergic airway inflammation. Previous results have shown that CD38, involved in generating calcium mobilizing molecules, contributes to airway hyperresponsiveness. [77–79]. Therapeutic strategies that target both CD38 and these miRNA networks may prove effective in reversal of allergen-induced changes in airway hyperresponsiveness and airway inflammation, particularly in the asthmatic patient.

## Supporting Information

S1 Data Complete list of differentially expressed genes in HASM cells transfected with a miR-708 mimic. Table shows fold-change in expression relative to expression in cells transfected with a scrambled control sequence and the p value.
(XLS)
